# Supplementary material for: Pupil size reveals arousal level fluctuations in human sleep
Source: Nat Commun. 2025 Feb 28;16:2070. doi: 10.1038/s41467-025-57289-5 (PMC11871316; doi:10.1038/s41467-025-57289-5)
Supplement: Supplementary file 1 — Supplementary Information [file 41467_2025_57289_MOESM1_ESM.pdf]

Supplementary Information

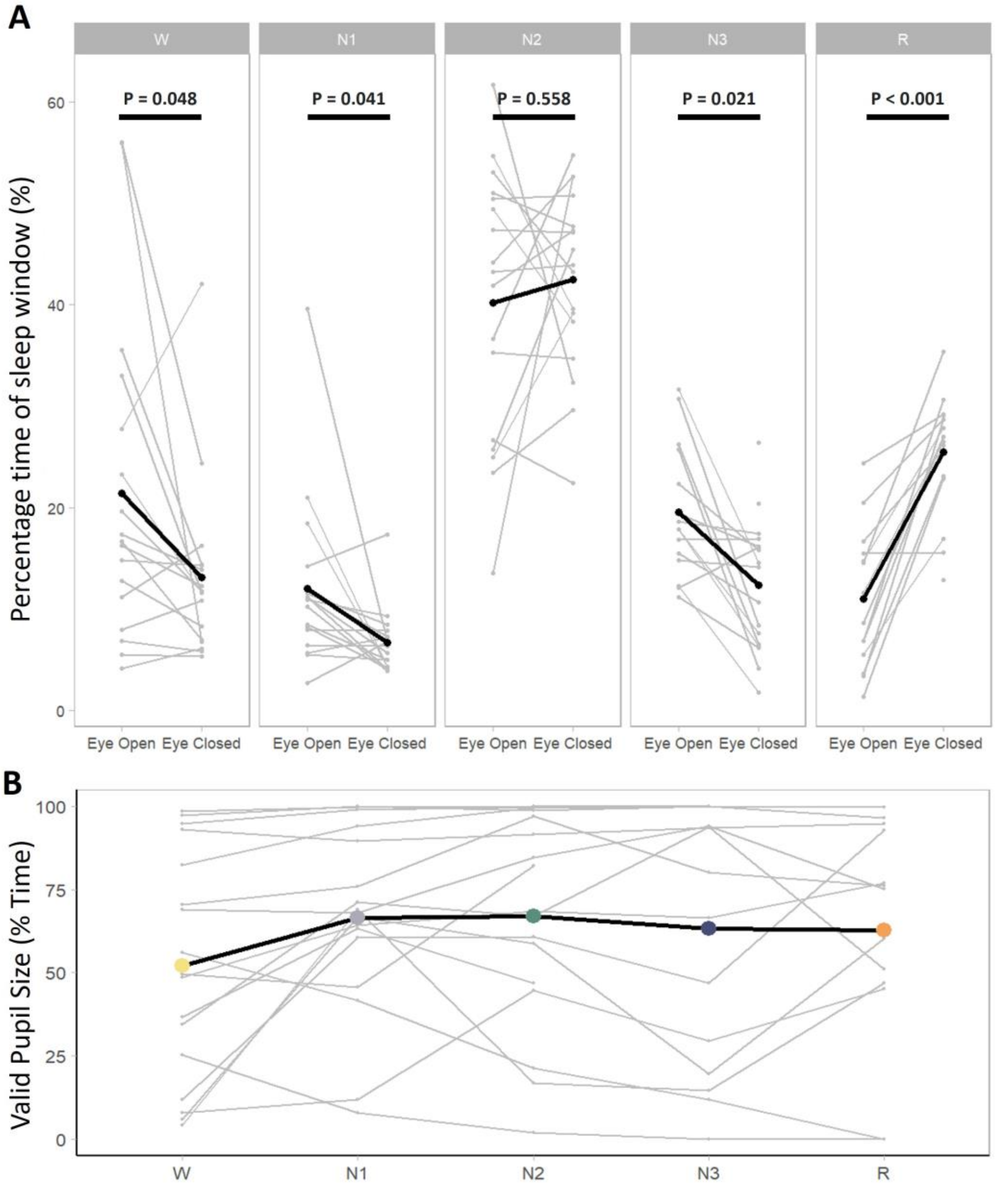

Supplementary Figure 1. Overnight sleep and pupil quality. (**A**) Percentage of time spent in each sleep stage in the sleep window when the right eye is taped open (Eye Open) versus in the sleep window after the tape is removed and the eye can be closed (Eye Closed). N1: NREM stage 1; N2: NREM stage 2; N3: NREM stage 3; R: rapid eye movement (REM). $t_{W}(16)=-2.259$, -8.36±3.70%, p=0.048, 95% CID (-15.82, -0.91), $t_{N1}(16)=-2.482$, -5.37±2.16%, p=0.041, 95% CID (-9.65, -1.09), $t_{N2}(16)=0.598$, 2.25±3.76%, p=0.558, 95% CID (-5.30, 9.79), $t_{N3}(12.23)=-3.129$, -7.23±2.31%, p=0.021, 95% CID (-11.75, -2.70), $t_{R}(13.87)=7.351$, 14.63±1.99%, p=<0.001, 95% CID (10.51, 18.58). p-values are based on post-hoc t-test and are adjusted for multiple comparisons using Benjamini-Hochberg correction. (**B**) Percentage of time during eye tracking with valid pupil size measurements for each sleep stage. W: wakefulness (yellow); N1: NREM stage 1 (gray); N2: NREM stage 2 (green); N3: NREM stage 3 (blue); R: rapid eye movement (REM, orange). The participant showing the most data loss across sleep stages (lowest and flattest gray line) was only included for pupil-related analyses during wake (Figure 1F, Figure 1I, and Supplementary Figure 2).

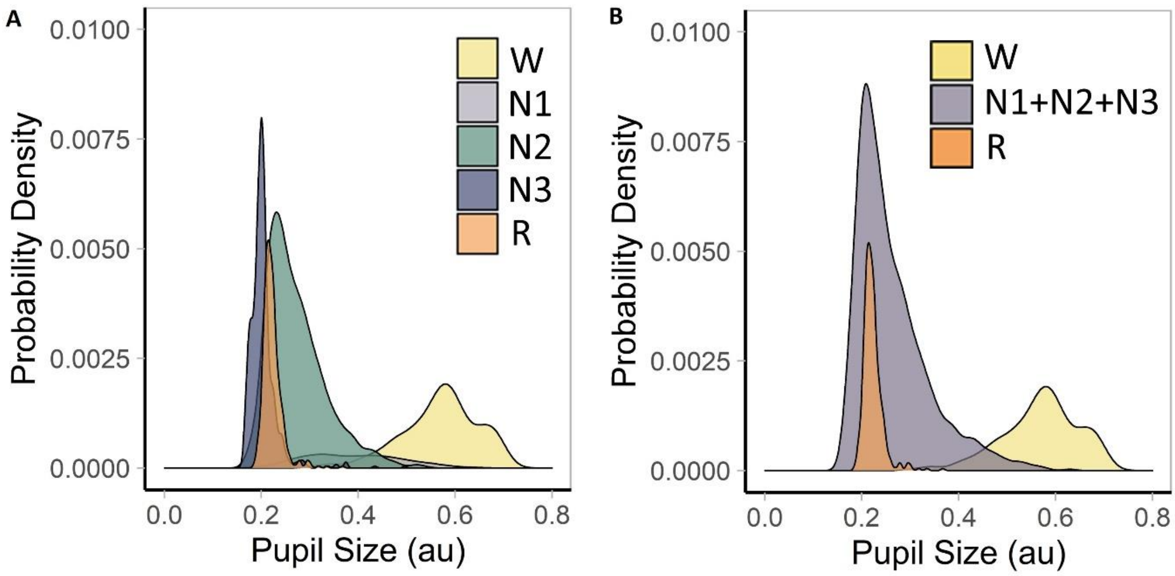

Supplementary Figure 2. Probability density plot of pupil size. (**A**) Pupil size probability for each sleep stage. (**B**) Pupil size probability for each sleep stage but with NREM sleep stages pooled together (N1+N2+N3). W: wakefulness (yellow); N1: NREM stage 1 (gray); N2: NREM stage 2 (green); N3: NREM stage 3 (blue); R: rapid eye movement (REM, orange), N1+N2+N3: NREM stages 1-3 pooled together (dark gray).

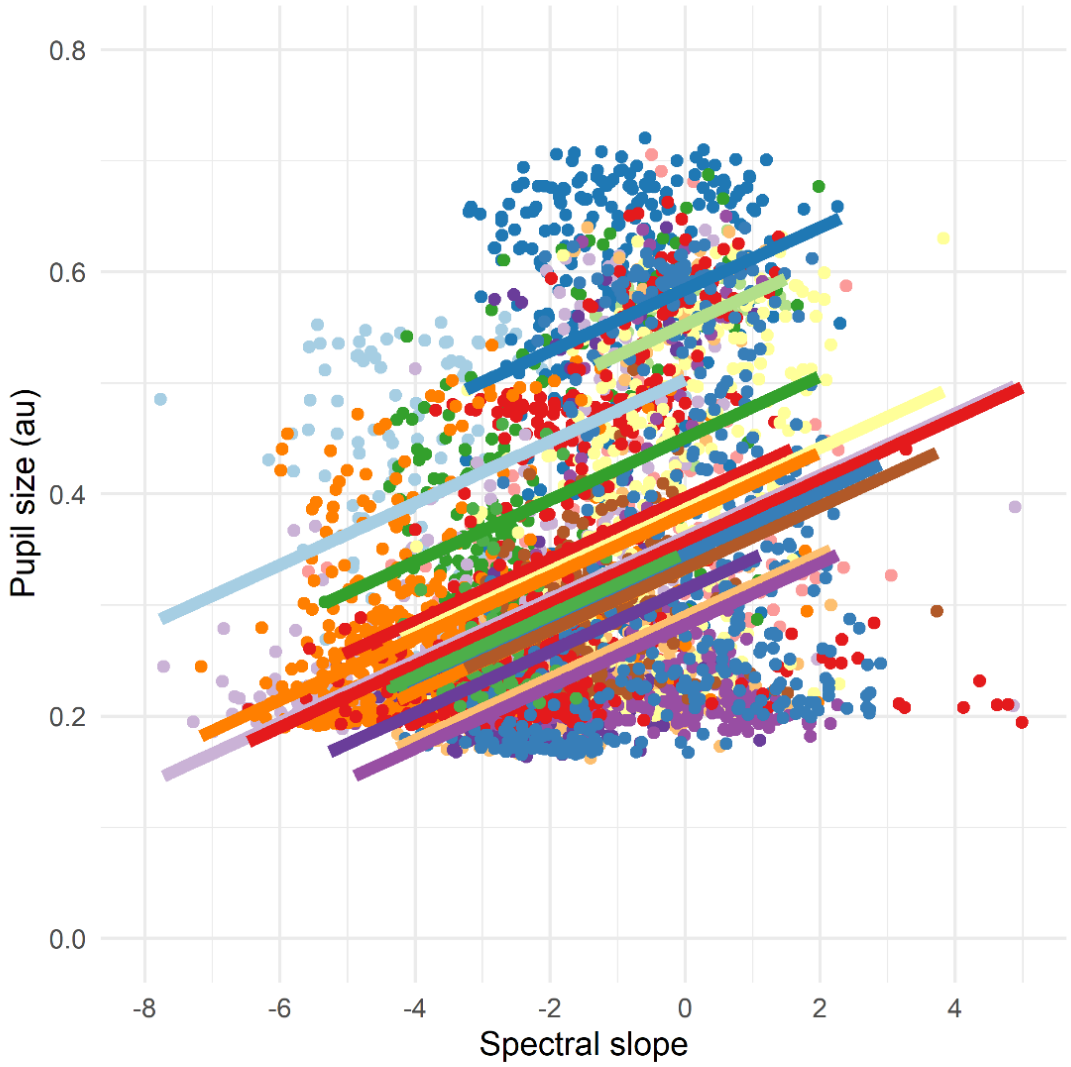

Supplementary Figure 3. Pupil size versus spectral slope for all sleep stages. Repeated measures correlation of pupil size versus spectral slope for all sleep stages: R=0.34, p<0.001, N=17. Different colors represent different participants.

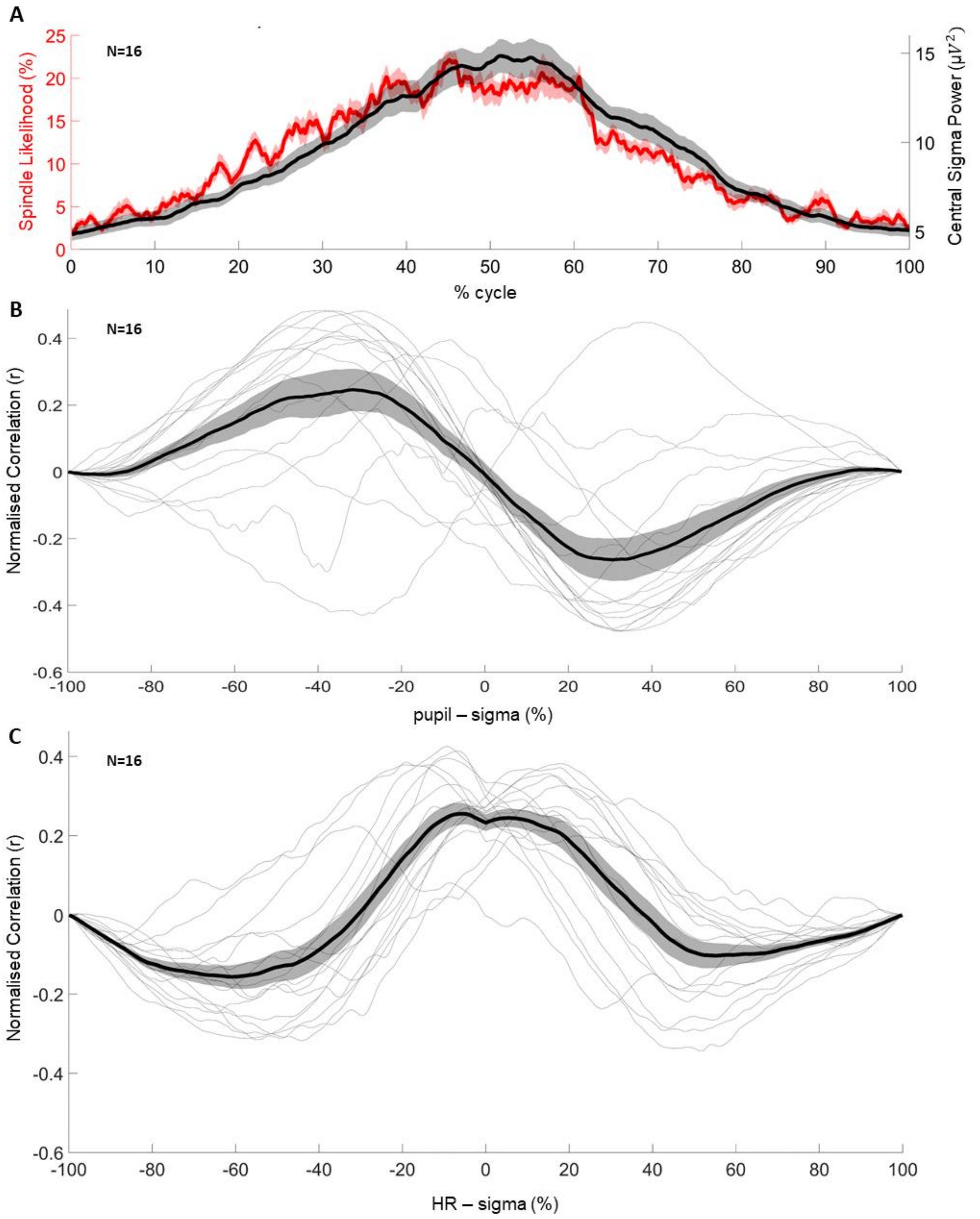

Supplementary Figure 4. Likelihoods of spindles during infraslow cycles and cross-correlations. (**A**) Spindle likelihood (red & left axis) and sigma power (black & right axis) normalized to the timing of the troughs of detected spindle infraslow fluctuations (ISF) during N2. (**B**) Cross-correlation of sigma power (source) and pupil size with the lag being with respect to the cycle percentage of the ISF. (**C**) Cross-correlation of sigma power (source) and heart rate with the lag being with respect to the cycle percentage of the ISF. Centre lines plots represent the mean across participants and shadings around center lines represent the standard error of the mean (SEM).

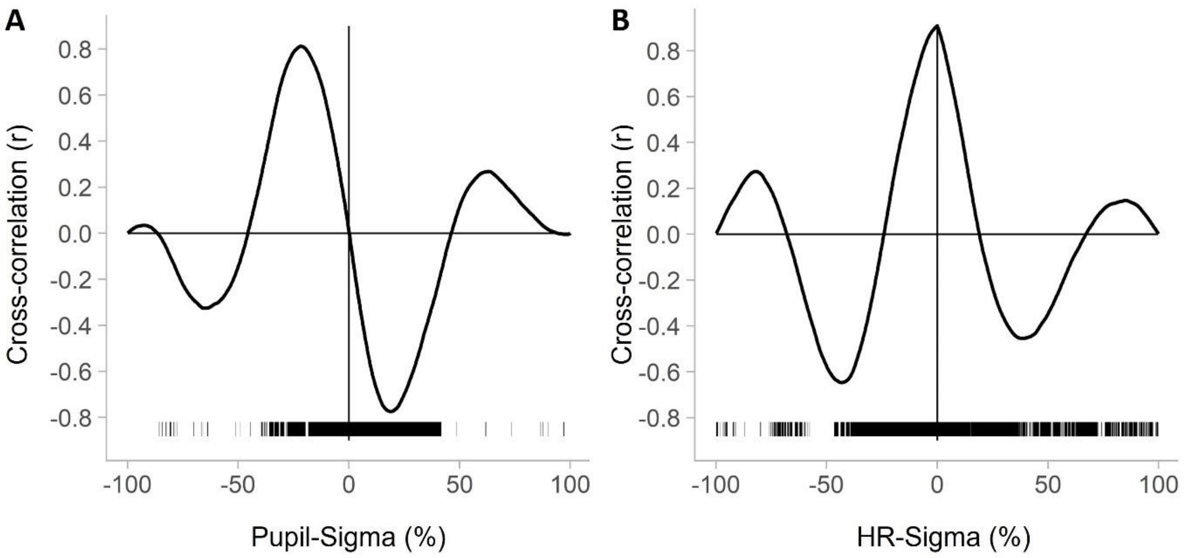

Supplementary Figure 5. Significance of cross-correlations during N2. (**A**) Cross-correlation of mean sigma power (source) and mean pupil size across participants (black line) with the lag being with respect to the cycle percentage of the ISF. (**B**) Cross-correlation of mean sigma power (source) and mean heart rate across participants (black line) with the lag being with respect to the cycle percentage of the ISF. We used the package *testcorr* in R, which implements robust procedures for testing the significance of the cross-correlation in bivariate data. The null hypothesis is that there is no correlation between the two signals being compared. The robust p-values below 0.001, indicating significant correlations are reported in the form of black horizontal lines at the bottom of each figure.

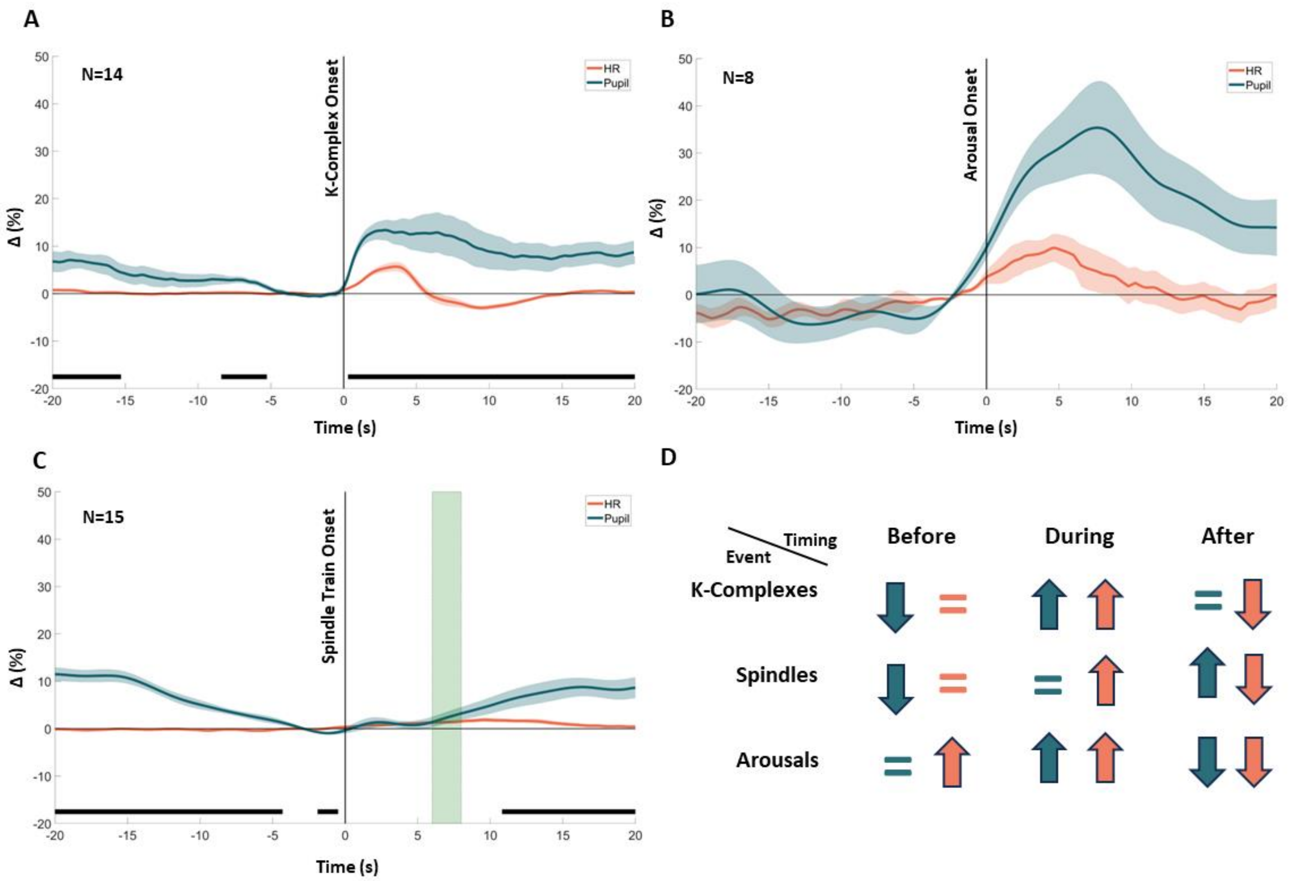

Supplementary Figure 6. Pupil size compared to instantaneous heart rate dynamics to N2 events. Pupil size color-coded in blue and heart rate color-coded in orange normalized to the 5s prior the detected event: K-complexes (**A**), sleep arousals (**B**), and spindle trains (**C**). Centre lines represent the group mean and shading around the centre lines the SEM. Black bars mark significant ($p<0.05$) corrected paired t-tests corrected for multiple comparisons (Benjamin-Hochberg correction) between pupil and HR dynamics. Green vertical shading in (**C**) reflects the mean ± 1 SEM of the median spindle train end across participants. (**D**) Scheme summarizing the temporal changes in pupil size (blue) and heart rate (orange) during K-complexes, spindles, and sleep arousals. Up and down arrows represent increases and decreases, respectively, while equal signs represents no change in dynamics. (**A**) Significant differences between pupil and HR dynamics surrounding K-complexes are seen during the 20s after its onset and from 20s before up to 5s before. (**B**) No significant differences were found between pupil and HR dynamics surrounding sleep arousals. (**C**) Significant differences between pupil and HR dynamics surrounding spindle trains were present almost everywhere except during the spindle train.

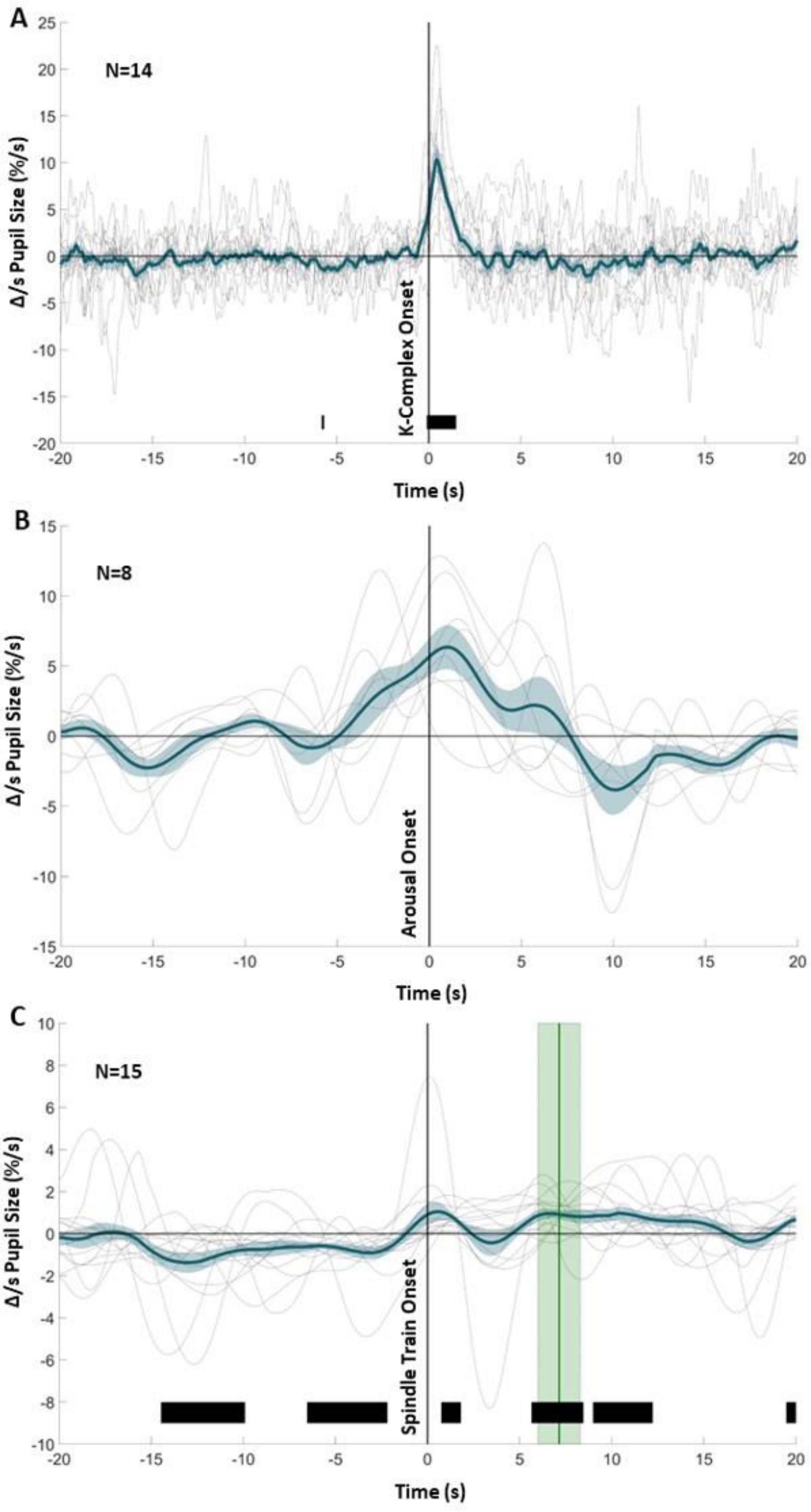

Supplementary Figure 7. Pupil size derivative during sleep events. (**A-C**) Derivative of pupil size that was normalized to the 5s prior the detected event in Figure 3A-C of the main manuscript: K-complexes, sleep arousals, and spindle trains (respectively). (**C**) Green vertical shading reflects the mean ± 1 SEM of the median spindle train end across participants. Blue centre lines represent the group mean and shading around the centre lines the SEM. Gray lines are the mean response of each participant. Black horizontal lines mark significant differences from zero *($p<0.05$).* p-values are based on post-hoc t-test and are adjusted for multiple comparisons using Benjamini-Hochberg correction.

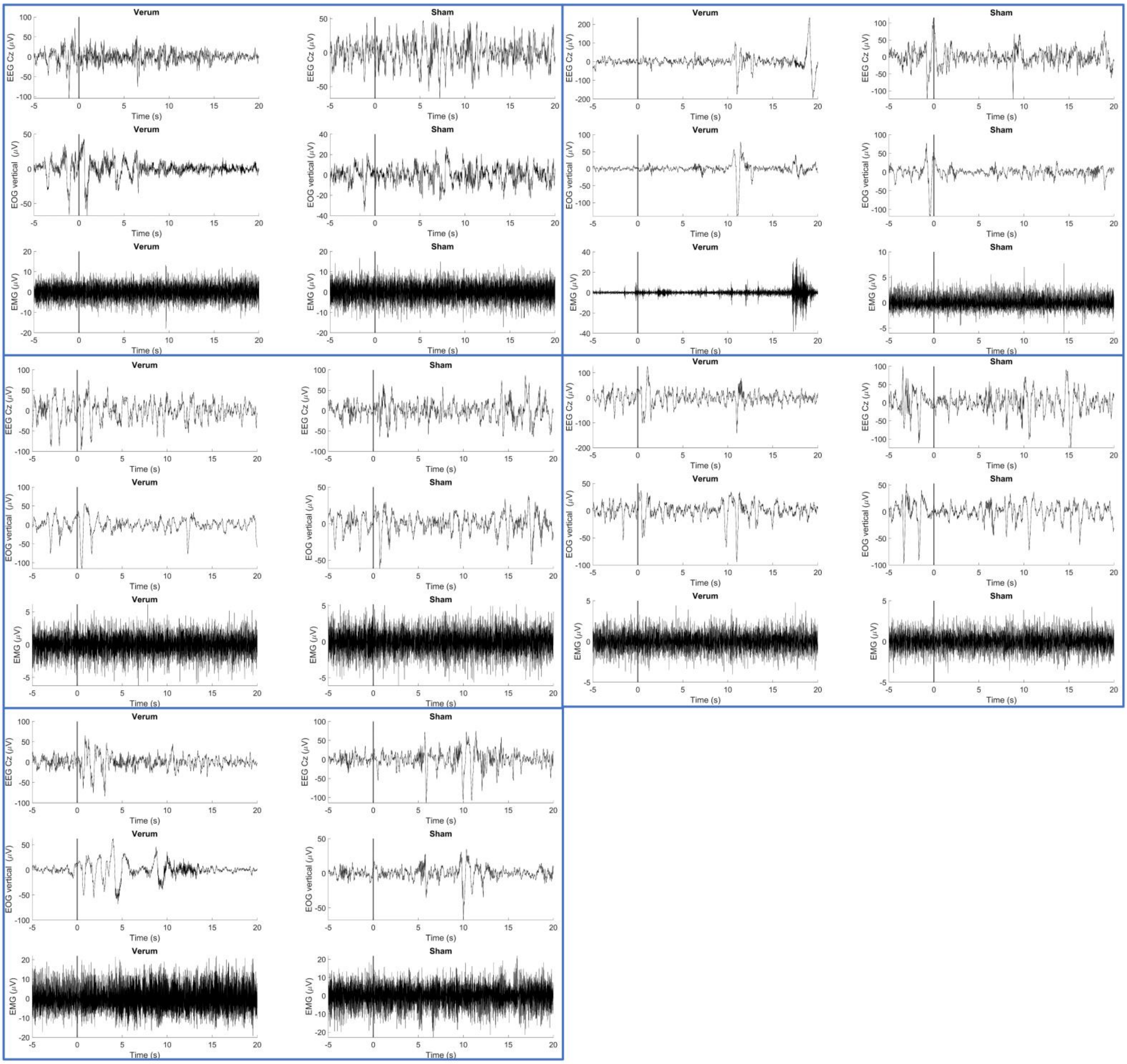

Supplementary Figure 8. Polysomnography data during stimulation windows. Example of polysomnography data during stimulation windows for both verum and sham trials in participants of stimulation protocol V1 that were included in the analysis reported in Figure 4, 5 in the main manuscript (see Supplementary Table 8 for details). Each blue quadrant contains an exemplary trial from the verum and sham from each participant. Data includes EEG, eye movements in vertical direction derived from EOG (EOG vertical) and EMG. EEG, EOG, and EMG signals were notch filtered at 50Hz and bandpass filtered in the 0.5 to 35Hz, 0.5 to 35Hz, and 10 to 90Hz frequency range (respectively) using MATLAB 2nd order Butterworth filters. Time zero is the detection of the stimulation window.

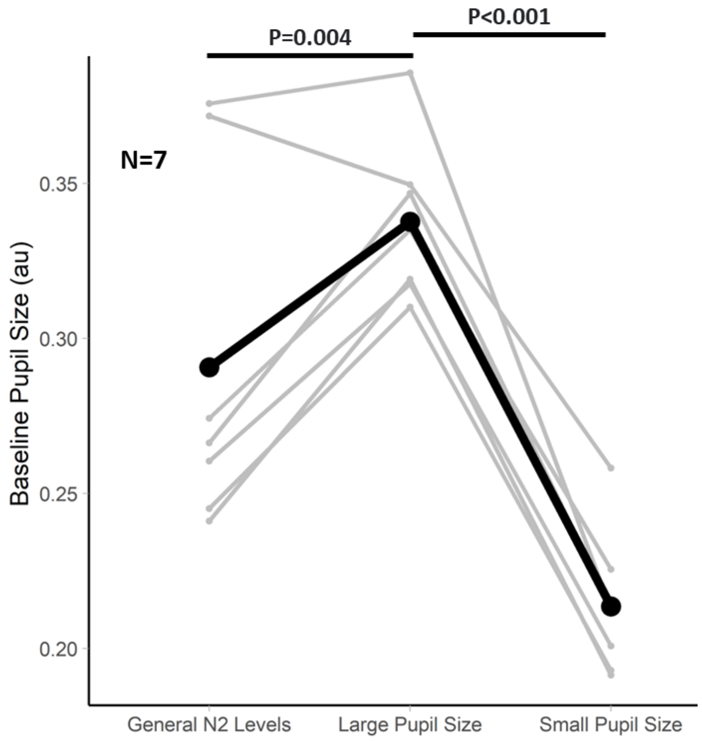

Supplementary Figure 9. Pupil size prior to stimulation versus general N2 levels. Pupil size during N2 compared to large pupil size at baseline prior to stimulation and small pupil size at baseline prior to stimulation. Post-hoc t-tests corrected for multiple comparisons revealed that large pupil size during Stimpre was greater than general N2 levels (t(16.3)= 3.37, 18.54±5.50%, p=0.004, 95% CID (0.01, 0.08)) and small pupil size baselines were smaller than general N2 levels (t(16.3)=-5.527, -25.21±3.40%, p<0.001, 95% CID (-0.11, -0.04)

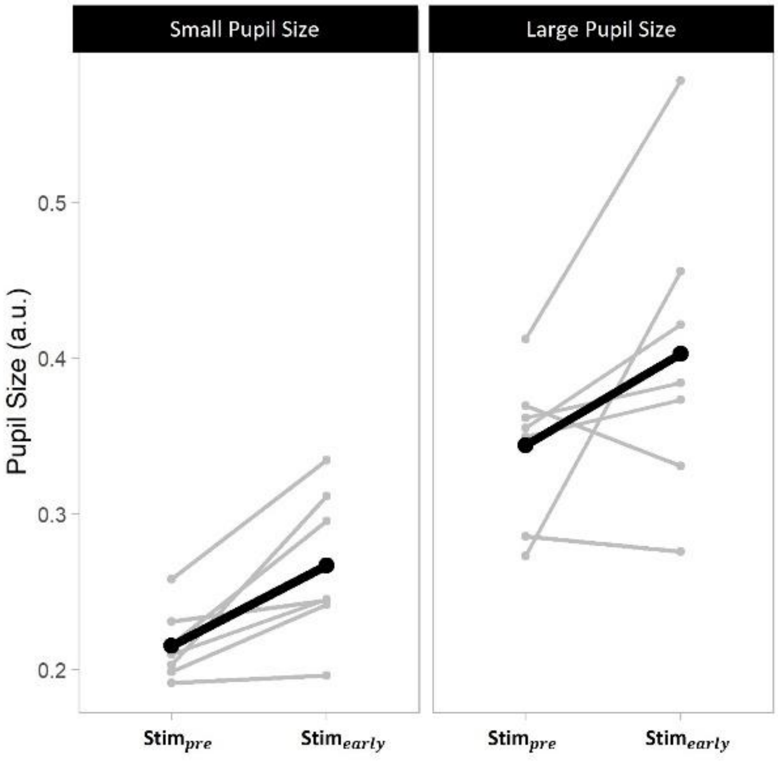

Supplementary Figure 10. Evoked pupil response for small and large pupil size at baseline. Pupil size 5s before ($Stim_{pre}$) and during the first 5s ($Stim_{early}$) of verum stimulation of trials with large (highest quartile) and small pupil baselines (lowest quartile)

to group into high and low arousal level trials. We found found main effects of pupil size at baseline ($F(1, 21)=54.94$, $p<0.001$) and evoked pupil dilation ($F(1, 21)=9.60$, $p=0.005$) but no interaction effect $F_{baselineXdilation}(1, 21)=0.042$, $p=0.829$.

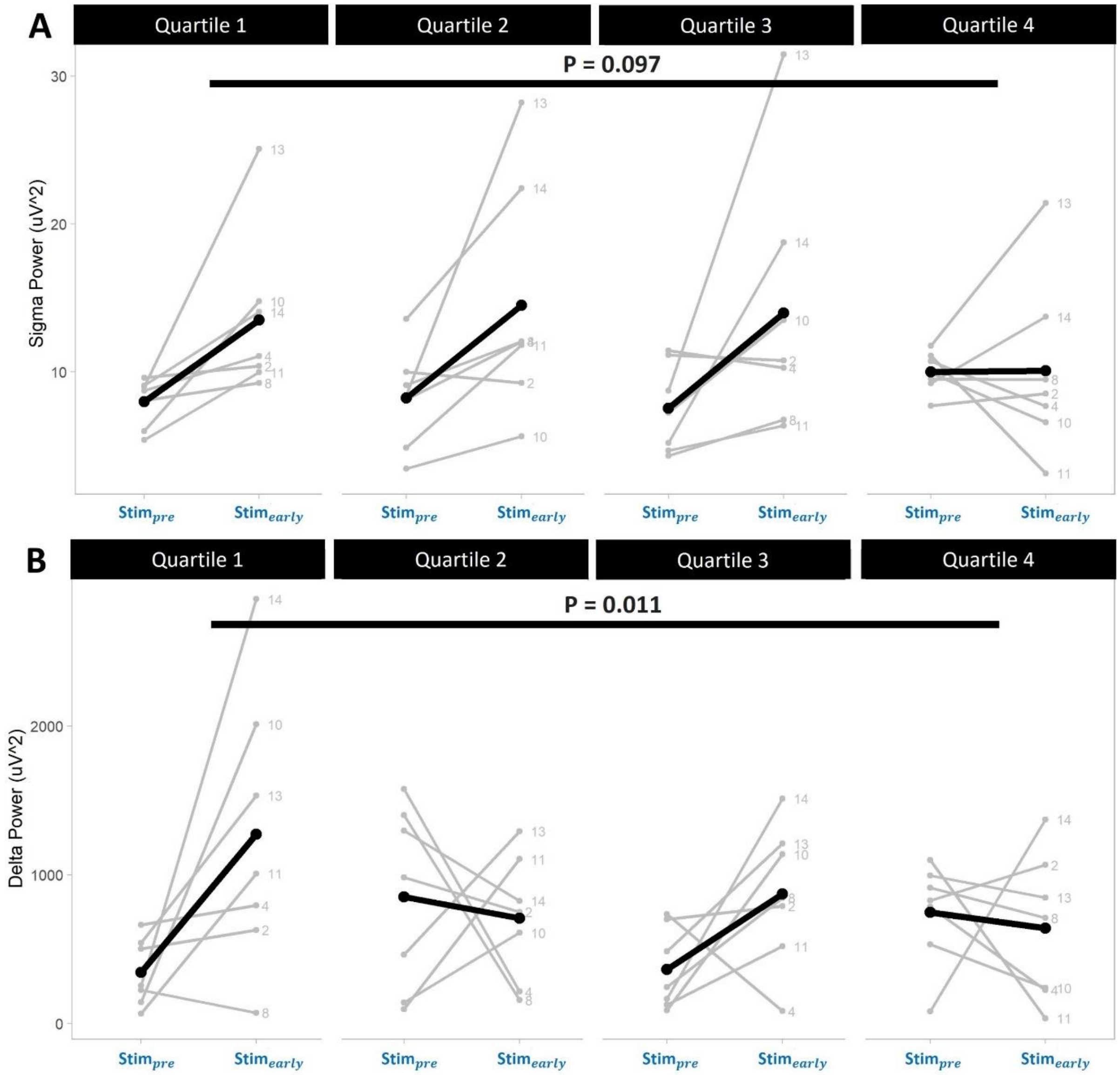

Supplementary Figure 11. EEG responses to auditory stimulation at pupil size baselines separate into quartiles. (A) Sigma power 5s before (Stim$_{pre}$) and during the first 5s (Stim$_{early}$) of verum stimulation trials sorted from trials with smallest pupil size at Stim$_{pre}$ (quartile 1) to trials with largest pupil size at Stim$_{pre}$ (quartile 4). Note that here quartile 1 and quartile 4 correspond to small and large quartiles in figure 5A in the main manuscript. We found no interaction effect between pupil baselines and sigma power at Stim$_{pre}$ and Stim$_{early}$, $F_{sigmaXpupilrange}(3, 49)=2.23$, $p=0.097$, but did find a main effect of sigma power changes from Stim$_{pre}$ to Stim$_{early}$, $F_{sigma}(1, 49)=20.43$, $p<0.001$. Every quartile except quartile 4, showed increased sigma power at Stim$_{early}$ compared to Stim$_{post}$. (**B**) Delta power 5s before (Stim$_{pre}$) and during the first 5s (Stim$_{early}$) of verum stimulation trials sorted from trials with smallest pupil size at Stim$_{pre}$ (quartile 1) to trials with largest pupil size at Stim$_{pre}$ (quartile 4). Note that here quartile 1 and quartile 4 correspond to small and large quartiles in figure 5B in the main manuscript. We found an interaction effect indicating varying evoked responses in delta power from Stim$_{pre}$ to Stim$_{early}$ in across pupil baseline conditions, $F_{deltaXpupilrange}(3, 49)=4.15$, $p=0.011$. For posthoc analysis we quantified the evoked response as the difference between Stim$_{early}$ and Stim$_{pre}$ and derived post-hoc p-values on the differences in evoked response between different pupil baseline conditions using Satterthwaite's method from the R package lmertest and corrected for multiple comparisons with the Hochberg method using the R package emmeans. Post-hoc analysis showed significant differences in evoked responses between quartile 1 and quartile 2 ($t(24.5)=2.97$, $p=0.040$), and between quartile 1 and quartile 4 ($t(24.5)=2.87$, $p=0.042$). No other contrasts were significant ($|t(24.5)|\leq1.80$, $p\geq0.306$). The number next to the gray lines correspond to the participant number. The stimulation protocol V0 was used in 2 and 4, and V1 for the rest. The horizontal black lines connecting pupil size ranges are the interaction effects between pupil size range and Stim$_{pre}$-Stim$_{early}$ power values. p-values are based on post-hoc t-test and are adjusted for multiple comparisons using Benjamini-Hochberg correction.

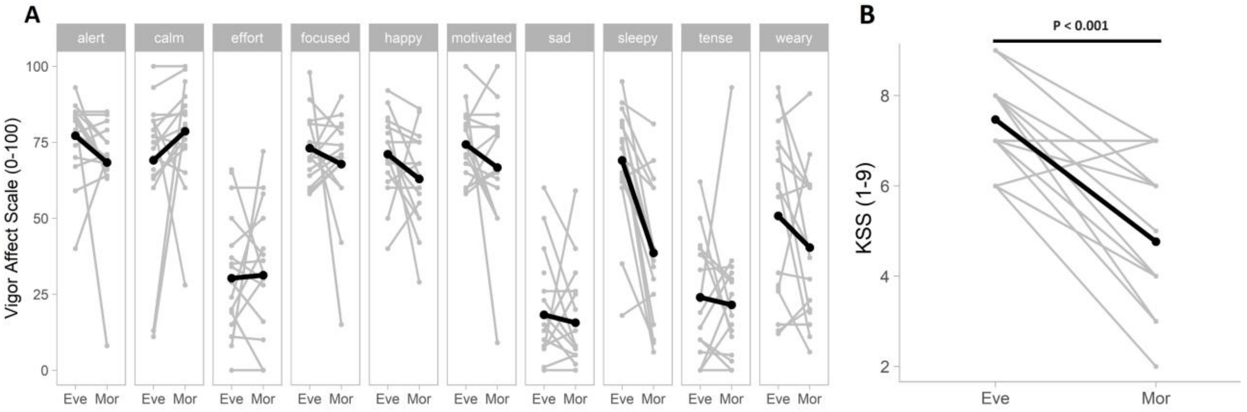

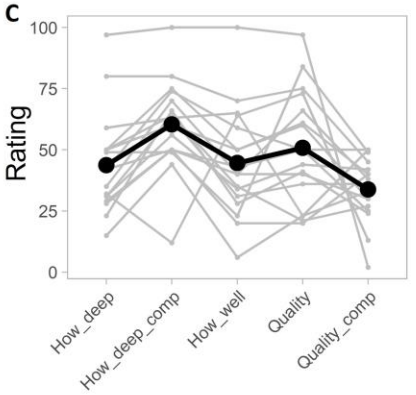

Acronym	Question	Lowest Value (=0)	Highest Value (=100)
How_deep	Wie tief haben Sie geschlafen?	sehr tief	sehr oberflächlich
	How deeply did you sleep?	very deep	very superficial
How_deep_comp	Wie war die Schlaftiefe im Vergleich zu Ihrer durchschnittlichen Schlaftiefe?	tiefer	oberflächlicher
	How was the depth of your sleep compared to your average sleep depth?	deeper	more superficial
How_well	Wie haben Sie geschlafen?	sehr ruhig	überhaupt nicht ruhig
	How did you sleep?	very calm	not calm at all
Quality	Wie würden Sie die Schlafqualität im Allgemeinen einschätzen?	sehr gut	sehr schlecht
	How would you rate your overall sleep quality?	very good	very bad
Quality_comp	Wie war die Schlafqualität im Vergleich zu Ihrer durchschnittlichen Schlafqualität?	schlechter	besser
	How was the sleep quality compared to your average sleep quality?	worse	better

Supplementary Figure 12. Vigor Affect and Sleep quality questionnaires. (**A**) Results for each dimension of the vigor affect questionnaire. One participant has missing data in the morning due to a measurement error. (**B**) Results for the Karolinska Sleepiness Scale (KSS), a more standard approach to assess sleepiness as compared to the sleepy dimension of the vigor affect scale. We found a main time of day effect indicating a decreased KSS score from Evening (Eve) to Morning (Mor) (-2.71±0.44; $F(1, 17)=38.11$, $p<0.001$). Gray lines are the mean response of each participant and black lines are the mean response across participants. (**C**) Left: Results for rating of sleep quality questions. Right: Table which questions each acronym along the x-axis in the right figure corresponds to in German (white rows) and English (gray rows).

DeepLabCut-based pupil size tracking versus established eye tracking

One participant sat alone in a room in a comfortable chair with their chin placed in a chin rest to ensure a stable head position. Their eyes were ~65 cm away from the eye tracker (Tobii TX300, Tobii Technology) that was positioned below the screen (240B7QPJ, resolution: 1,680 × 1,050 pixels; Philips) to allow for optimal eye tracking and measurement of pupil size. The participant was instructed to look at the fixation dot displayed in the center of the screen while the background display changed to one of three light intensities (darker, neutral, brighter) in a counterbalanced and randomized order, thereby inducing pupillary light reflexes at varying intensities. Pupil diameter for the right eye was recorded using the Tobii TX300 SDK for MATLAB v.3 and MATLAB 2013a). At the same time, the right eye was tracked using the same Pupil Core goggles used in the main study and Pupil Capture v3.4.0 software (Pupil Labs GmbH, Berlin, Germany). Using the video from Pupil Capture, pupil size for the right eye was calculated exactly as described in line XXX in the methods section. Pupil size derived from both eye trackers were down sampled to 25Hz, synchronized, normalized using z-scoring, and overlayed as shown in Supplementary Figure 13A. Pupil size derived using both methods were very strongly correlated ($R_{pearson}$=0.995, p<0.001, Supplementary Fig. 13B). The main limitation of this comparison is the varying intensity of infrared light emitted by the Tobii TX300. During the measurement, this varying intensity caused overexposure in the Pupil Core infrared cameras. As a result, the video footage became bleached, preventing the DeepLabCut algorithm from accurately tracking the anatomical features needed to measure pupil size. This is reflected in the missing orange values in Supplementary Figure 13A.

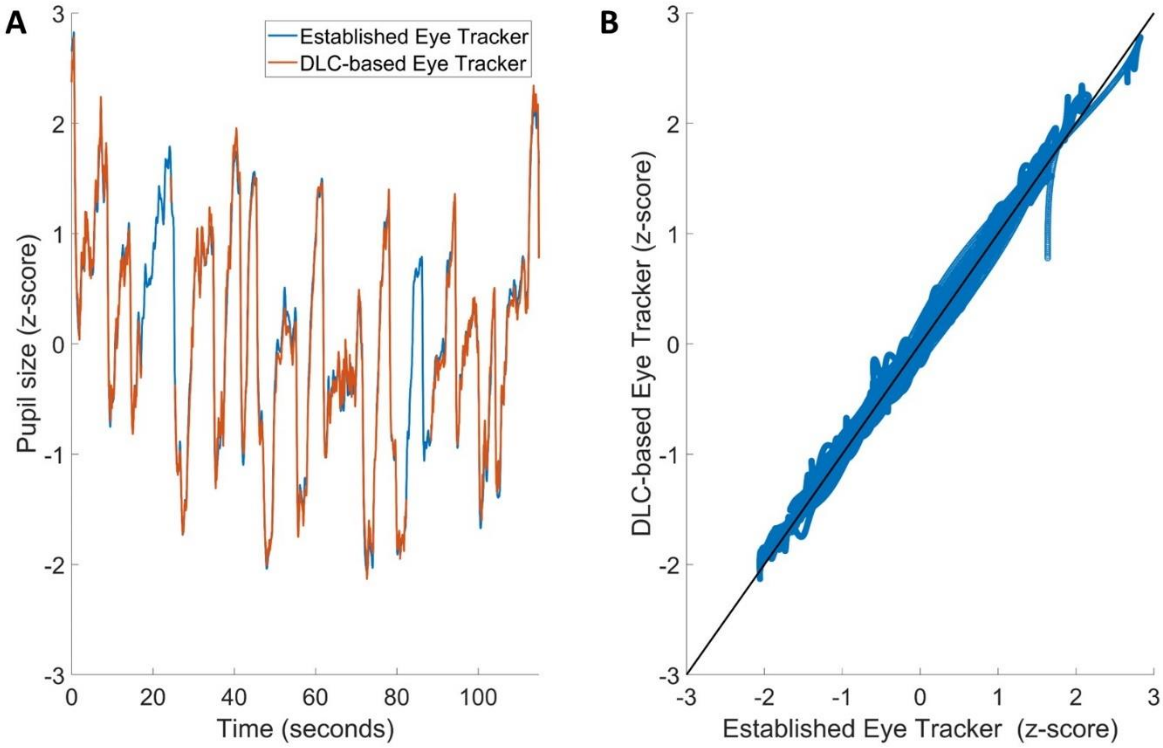

Supplementary Figure 13. DeepLabCut-based pupil size tracking versus established eye tracking. (**A**) Pupil size z-scored independently for both the established eye tracker assessment (blue) and the overlayed DeepLabCut (DLC)-based assessment (orange). (**B**) Pearson correlation of both eye tracking assessments. The black line indicates identical pupil size measurements with both assessments (N=1).

Supplementary Table 1. Pupil size contrasts across sleep stages. P-values are those shown in Figure 1F.

Effect	EMMeans	df	t.ratio	95% CI (Lower)	95% CI (Upper)	p-value	Effect size (Cohen's D)
AWAKE - NREM1	0.156	60.387	10.682	0.114	0.199	<0.001	3.94
AWAKE - NREM2	0.267	60.75	18.256	0.225	0.31	<0.001	6.73
AWAKE - NREM3	0.326	62.898	20.921	0.281	0.372	<0.001	8.23
AWAKE - REM	0.316	64.131	19.189	0.268	0.364	<0.001	7.97
NREM1 - NREM2	0.111	60.75	7.58	0.068	0.154	<0.001	2.8
NREM1 - NREM3	0.17	62.898	10.91	0.125	0.216	<0.001	4.29
NREM1 - REM	0.16	64.131	9.707	0.112	0.208	<0.001	4.03
NREM2 - NREM3	0.059	61.443	3.824	0.014	0.104	0.001	1.49
NREM2 - REM	0.049	62.641	2.994	0.001	0.097	0.008	1.24
NREM3 - REM	-0.01	60.699	-0.607	-0.06	0.039	0.546	-0.26

Supplementary Table 2. Spectral slope contrasts across sleep stages. P-values are those shown in Figure 1G.

Effect	EMMeans	df	t.ratio	95% CI (Lower)	95% CI (Upper)	p-value	Effect size (Cohen's D)
Wake - N1	0.585	72.25	3.292	0.07	1.099	0.005	1.16
Wake - N2	1.163	72.25	6.548	0.649	1.678	<0.001	2.32
Wake - N3	1.594	72.25	8.974	1.08	2.109	<0.001	3.17
Wake - REM	1.961	72.25	11.04	1.447	2.476	<0.001	3.9
N1 - N2	0.578	72.25	3.256	0.064	1.093	0.005	1.15
N1 - N3	1.009	72.25	5.682	0.495	1.524	<0.001	2.01
N1 - REM	1.377	72.25	7.748	0.862	1.891	<0.001	2.74
N2 - N3	0.431	72.25	2.426	-0.083	0.946	0.036	0.86
N2 - REM	0.798	72.25	4.492	0.284	1.313	<0.001	1.59
N3 - REM	0.367	72.25	2.066	-0.147	0.882	0.042	0.73

Supplementary Table 3. Heart rate contrasts across sleep stages. P-values are those shown in Figure 1H.

Effect	EMMeans	df	t.ratio	95% CI (Lower)	95% CI (Upper)	p-value	Effect size (Cohen's D)
AWAKE - NREM1	5.983	59.388	4.093	1.721	10.244	0.001	1.51
AWAKE - NREM2	6.914	59.534	4.712	2.636	11.191	<0.001	1.75
AWAKE - NREM3	5.323	59.837	3.376	0.728	9.919	0.01	1.35
AWAKE - REM	2.936	59.956	1.758	-1.931	7.803	0.419	0.74
NREM1 - NREM2	0.931	59.534	0.634	-3.347	5.208	0.677	0.24
NREM1 - NREM3	-0.66	59.837	-0.419	-5.255	3.936	0.677	-0.17
NREM1 - REM	-3.047	59.956	-1.824	-7.914	1.821	0.419	-0.77
NREM2 - NREM3	-1.591	59.573	-1.022	-6.13	2.949	0.677	-0.4
NREM2 - REM	-3.977	59.695	-2.412	-8.784	0.829	0.133	-1.01
NREM3 - REM	-2.387	59.421	-1.406	-7.337	2.563	0.66	-0.6

Supplementary Table 4. Pupil size contrasts across phases of sigma power ISFs. P-values are those shown in Figure 2G.

Effect	EMMeans	df	t.ratio	95% CI (Lower)	95% CI (Upper)	p-value	Effect size (Cohen's D)
Rise - Peak	-0.011	51.2	-2.271	-0.024	0.002	0.058	-0.83
Rise - Fall	-0.026	51.2	-5.482	-0.04	-0.013	<0.001	-2
Rise - Trough	-0.011	51.2	-2.247	-0.024	0.002	0.058	-0.82
Peak - Fall	-0.016	51.2	-3.212	-0.029	-0.002	0.009	-1.17
Peak - Trough	0	51.2	0.024	-0.013	0.013	0.981	0.01
Fall - Trough	0.016	51.2	3.235	0.002	0.029	0.009	1.18

Supplementary Table 5. Heart rate contrasts across phases of sigma power ISFs. P-values are those shown in Figure 2H.

Effect	EMMeans	df	t.ratio	95% CI (Lower)	95% CI (Upper)	p-value	Effect size (Cohen's D)
Rise - Peak	-0.854	51.2	-3.015	-1.632	-0.077	0.012	-1.1
Rise - Fall	-0.106	51.2	-0.376	-0.884	0.671	0.709	-0.14
Rise - Trough	1.036	51.2	3.657	0.258	1.813	0.002	1.34
Peak - Fall	0.748	51.2	2.64	-0.03	1.525	0.022	0.96
Peak - Trough	1.89	51.2	6.672	1.112	2.667	<0.001	2.44
Fall - Trough	1.142	51.2	4.032	0.365	1.92	0.001	1.47

Supplementary Table 6. K-complex likelihood contrasts across phases of sigma power ISFs. P-values are those shown in Figure 2I.

Effect	EMMeans	df	t.ratio	95% CI (Lower)	95% CI (Upper)	p-value	Effect size (Cohen's D)
Rise - Peak	-2.629	51.2	-5.599	-3.917	-1.34	<0.001	-2.04
Rise - Fall	-2.225	51.2	-4.74	-3.514	-0.937	<0.001	-1.73
Rise - Trough	-0.228	51.2	-0.486	-1.517	1.06	0.629	-0.18
Peak - Fall	0.403	51.2	0.859	-0.885	1.692	0.629	0.31
Peak - Trough	2.4	51.2	5.112	1.112	3.689	<0.001	1.87
Fall - Trough	1.997	51.2	4.254	0.708	3.286	<0.001	1.55

Supplementary Table 7. Pupil size and heart rate during the 5s prior to N2 events versus general N2 pupil size. Linear mixed-effects models showing significance for the fixed effect (two-sided, Pupil: $F(3, 39.30)=3.68$, $p=0.020$; Heart rate: $F(3, 40.03)=6.34$, $p=0.001$), we derived post-hoc p-values using Satterthwaite's method and corrected for multiple comparisons with the Hochberg method.

Metric	5s prior N2 events vs general N2	Mean % change	SEM % change	t	df	p-value
Pupil size	K-complex - N2	-8.45	2.33	-2.220	41.8	0.0638
	Arousals- N2	0.95	4.94	0.380	43.3	0.7061
	Spindles - N2	-8.34	2.75	-2.300	41.5	0.0638
Heart rate	K-complex - N2	-1.18	0.46	-1.253	43.2	0.381
	Arousals- N2	3.50	1.59	3.200	43.4	0.008
	Spindles - N2	-0.89	0.71	-0.885	43.2	0.381

Supplementary Table 8. Stimulation protocols. Description of what participants were included in each stimulation protocol and corresponding analysis in Figure 4 and Figure 5. The number of sham and verum windows and the number of sleep arousals co-occurring with these windows are reported.

Participant number	Stimulation protocol	Stimulation analysis included in	Number of sham windows	Number of verum windows	Number of arousals during sham windows	Number of arousals during verum windows	Sham windows with arousals (%)	Verum windows with arousals (%)
1	V0	None	-	0	-	0	-	0
2	V0	Figure 5 All	-	303	-	4	-	0.33
3	V0	None	-	85	-	3	-	1.17
4	V0	Figure 5 All	-	94	-	1	-	1.06
5	V0.5	None	12	14	0	0	0	0
6	V0.5	None	0	0	0	0	NaN	NaN
7	V0.5	None	1	1	0	0	0	0
8	V1	Figure 4 All and Figure 5 All	74	72	1	1	1.35	1.39
9	V1	Figure 4 All	15	14	0	0	0	0
10	V1	Figure 4 All and Figure 5 All	66	64	0	0	0	0
11	V1	Figure 4 All and Figure 5 All	46	45	0	0	0	0
12	V1	Figure 4 All	56	55	0	1	0	1.82
13	V1	Figure 4 All and Figure 5 All	48	44	0	0	0	0
14	V1	Figure 4 All and Figure 5 All	47	45	0	0	0	0
15	V1	Figure 4 All	55	54	0	0	0	0
16	V1	Figure 4 B,C,E,F	15	15	1	0	6.67	0
17	V1	Figure 4 B,C,E,F	8	7	0	0	0	0
