# Supplementary material for: Pupil size reveals arousal level fluctuations in human sleep
Source: Nat Commun. 2025 Feb 28;16:2070. doi: 10.1038/s41467-025-57289-5 (PMC11871316; doi:10.1038/s41467-025-57289-5)
Supplement: Supplementary file 2 — Description of Additional Supplementary Files [file 41467_2025_57289_MOESM2_ESM.pdf]

Description of Additional Supplementary Files

Supplementary Movie 1: Pupil size change in response to auditory stimulation during NREM sleep. Top to bottom: video stream from eye tracker, onset of tones, EEG channel FPz, EEG channel Cz, and pupil size changes. Units in X-axis are seconds
