# Supplementary material for: Pupil size reveals arousal level fluctuations in human sleep
Source: Nat Commun. 2025 Feb 28;16:2070. doi: 10.1038/s41467-025-57289-5 (PMC11871316; doi:10.1038/s41467-025-57289-5)
Supplement: Supplementary file 4 — Reporting Summary [file 41467_2025_57289_MOESM4_ESM.pdf]

nature portfolio

Corresponding author(s): Caroline Lustenberger

Last updated by author(s): Jan 14, 2025

Reporting Summary

Nature Portfolio wishes to improve the reproducibility of the work that we publish. This form provides structure for consistency and transparency in reporting. For further information on Nature Portfolio policies, see our Editorial Policies and the Editorial Policy Checklist.

Statistics

For all statistical analyses, confirm that the following items are present in the figure legend, table legend, main text, or Methods section.

n/a	Confirmed	
☐	☒	The exact sample size (n) for each experimental group/condition, given as a discrete number and unit of measurement
☐	☒	A statement on whether measurements were taken from distinct samples or whether the same sample was measured repeatedly
☐	☒	The statistical test(s) used AND whether they are one- or two-sided<br>*Only common tests should be described solely by name; describe more complex techniques in the Methods section.*
☐	☒	A description of all covariates tested
☐	☒	A description of any assumptions or corrections, such as tests of normality and adjustment for multiple comparisons
☐	☒	A full description of the statistical parameters including central tendency (e.g. means) or other basic estimates (e.g. regression coefficient) AND variation (e.g. standard deviation) or associated estimates of uncertainty (e.g. confidence intervals)
☐	☒	For null hypothesis testing, the test statistic (e.g. F, t, r) with confidence intervals, effect sizes, degrees of freedom and P value noted<br>*Give P values as exact values whenever suitable.*
☒	☐	For Bayesian analysis, information on the choice of priors and Markov chain Monte Carlo settings
☒	☐	For hierarchical and complex designs, identification of the appropriate level for tests and full reporting of outcomes
☐	☒	Estimates of effect sizes (e.g. Cohen's d, Pearson's r), indicating how they were calculated

Our web collection on statistics for biologists contains articles on many of the points above.

Software and code

Policy information about availability of computer code

Data collection	MATLAB (2019a), OpenVIBE (2.2.0), Pupil Capture
Data analysis	MATLAB (2019a), Python (3.8), DeepLabCut (2.2), R (3.6.3)

For manuscripts utilizing custom algorithms or software that are central to the research but not yet described in published literature, software must be made available to editors and reviewers. We strongly encourage code deposition in a community repository (e.g. GitHub). See the Nature Portfolio guidelines for submitting code & software for further information.

Data

Policy information about availability of data

All manuscripts must include a data availability statement. This statement should provide the following information, where applicable:

- Accession codes, unique identifiers, or web links for publicly available datasets
- A description of any restrictions on data availability
- For clinical datasets or third party data, please ensure that the statement adheres to our policy

Source data for the main figures in the manuscript and sample data to calculate pupil size from DeepLabCut markers are openly available in the ETH Zurich Research Collection, reference number TBD (currently generated by the ETH Research Collection and adapted in the proofs once available).

Research involving human participants, their data, or biological material

Policy information about studies with human participants or human data. See also policy information about sex, gender (identity/presentation), and sexual orientation and race, ethnicity and racism.

Reporting on sex and gender	During the recruitment process, participants' sex was self-reported. We did not conduct a sex-based analysis of our results due to insufficiently large sample size to enable meaningful conclusions. All participants provided written informed consent prior to study participation.
Reporting on race, ethnicity, or other socially relevant groupings	During the recruitment process, we did not ask volunteers to report their race, ethnicity, or other socially relevant information
Population characteristics	The 17 included participants (10 female, mean±sd age; 29.83±8.69 years) were non-smokers, reported a regular sleep-wake rhythm, and had a body-mass index between 17-30. Participants reported no presence of psychiatric/neurological diseases, sleep disorders, or clinically significant concomitant diseases.
Recruitment	18 participants were recruited using advertisements on different internet platforms. One participant was excluded because of predefined exclusion criteria, as they reported an elevated level of eye dryness (score greater than 12 in the Ocular Surface Disease Index). Participants received CHF70 for participation.
Ethics oversight	This study was approved by the Cantonal Ethics Committee Zurich (reference number: KEK ZH, BASEC 2022-00340

Note that full information on the approval of the study protocol must also be provided in the manuscript.

Field-specific reporting

Please select the one below that is the best fit for your research. If you are not sure, read the appropriate sections before making your selection.

[x] Life sciences [ ] Behavioural & social sciences [ ] Ecological, evolutionary & environmental sciences

For a reference copy of the document with all sections, see nature.com/documents/nr-reporting-summary-flat.pdf

Life sciences study design

All studies must disclose on these points even when the disclosure is negative.

Sample size	18: A formal sample size calculation was not conducted for this study. This sample size has been shown to provide sufficient statistical power in similar experimental designs with within-subject comparisons and is commonly used in the field to ensure robust and reproducible findings. Since there were no previous studies measuring pupil size during sleep, we based our sample size estimations on previous sleep studies showing a link between physiological activity and sleep macro- and microstructure.
Data exclusions	One participant was excluded because of predefined exclusion criteria, as they reported an elevated level of eye dryness (score greater than 12 in the Ocular Surface Disease Index).
Replication	This is the first study to report pupil size during sleep and replication is therefore not possible.
Randomization	Single condition, no randomization
Blinding	Single condition, no blinding

Reporting for specific materials, systems and methods

We require information from authors about some types of materials, experimental systems and methods used in many studies. Here, indicate whether each material, system or method listed is relevant to your study. If you are not sure if a list item applies to your research, read the appropriate section before selecting a response.

Materials & experimental systems

n/a	Involved in the study	
[x]	[ ]	Antibodies
[x]	[ ]	Eukaryotic cell lines
[x]	[ ]	Palaeontology and archaeology
[x]	[ ]	Animals and other organisms
[x]	[ ]	Clinical data
[x]	[ ]	Dual use research of concern
[x]	[ ]	Plants

Methods

n/a	Involved in the study	
[x]	[ ]	ChIP-seq
[x]	[ ]	Flow cytometry
[x]	[ ]	MRI-based neuroimaging

Plants

Seed stocks	*Report on the source of all seed stocks or other plant material used. If applicable, state the seed stock centre and catalogue number. If plant specimens were collected from the field, describe the collection location, date and sampling procedures.*
Novel plant genotypes	*Describe the methods by which all novel plant genotypes were produced. This includes those generated by transgenic approaches, gene editing, chemical/radiation-based mutagenesis and hybridization. For transgenic lines, describe the transformation method, the number of independent lines analyzed and the generation upon which experiments were performed. For gene-edited lines, describe the editor used, the endogenous sequence targeted for editing, the targeting guide RNA sequence (if applicable) and how the editor was applied.*
Authentication	*Describe any authentication procedures for each seed stock used or novel genotype generated. Describe any experiments used to assess the effect of a mutation and, where applicable, how potential secondary effects (e.g. second site T-DNA insertions, mosiacism, off-target gene editing) were examined.*
